# Chitosan and chitosan chlorhydrate based various approaches for enhancement of dissolution rate of carvedilol

**DOI:** 10.1186/2008-2231-20-93

**Published:** 2012-12-13

**Authors:** Amol S Shete, Adhikrao V Yadav, Srinivasa M Murthy

**Affiliations:** 1 Department of Pharmaceutics, Shree Santkrupa College of Pharmacy, Ghogaon, Tal. Karad Dist, Satara, MS, India; 2 Gourishankar Institute of Pharmaceutical Education and Research, Limb, Satara, 415002, India; 3 Vignan Institute of Pharmaceutical sciences, Deshmukhi, 508284, Nalgonda Dist, India

**Keywords:** Solubility, Dissolution, Carvedilol, Hydrosols, Chitosan and chitosan chlorhydrate

## Abstract

**Background and the purpose of the study:**

Carvedilol nonselective β-adrenoreceptor blocker, chemically (±)-1-(Carbazol-4-yloxy)-3-[[2-(o-methoxypHenoxy) ethyl] amino]-2-propanol, slightly soluble in ethyl ether; and practically insoluble in water, gastric fluid (simulated, TS, pH 1.1), and intestinal fluid (simulated, TS without pancreatin, pH 7.5) Compounds with aqueous solubility less than 1% W/V often represents dissolution rate limited absorption. There is need to enhance the dissolution rate of carvedilol. The objective of our present investigation was to compare chitosan and chitosan chlorhydrate based various approaches for enhancement of dissolution rate of carvedilol.

**Methods:**

The different formulations were prepared by different methods like solvent change approach to prepare hydrosols, solvent evaporation technique to form solid dispersions and cogrind mixtures. The prepared formulations were characterized in terms of saturation solubility, drug content, infrared spectroscopy (FTIR), differential scanning calorimetry (DSC), powder X-ray diffraction (PXRD), electron microscopy, *in vitro* dissolution studies and stability studies.

**Results:**

The practical yield in case of hydrosols was ranged from 59.76 to 92.32%. The drug content was found to uniform among the different batches of hydrosols, cogrind mixture and solid dispersions ranged from 98.24 to 99.89%. There was significant improvement in dissolution rate of carvedilol with chitosan chlorhdyrate as compare to chitosan and explanation to this behavior was found in the differences in the wetting, solubilities and swelling capacity of the chitosan and chitosan salts, chitosan chlorhydrate rapidly wet and dissolve upon its incorporation into the dissolution medium, whereas the chitosan base, less water soluble, would take more time to dissolve.

**Conclusion:**

This technique is scalable and valuable in manufacturing process in future for enhancement of dissolution of poorly water soluble drugs.

## Background

The rate of absorption and bioavailability of poorly water soluble drugs is often controlled by the rate of dissolution of the drug in gastrointestinal tract. Many technological methods of enhancing the dissolution characteristics of slightly water soluble drugs have been reported in literature. These include reducing particle size to increase the surface area [1], solublization in surfactant system [2], formation of water soluble complexes [3], use of prodrug, drug derivatization and manipulation of solid state of drug substance to improve drug dissolution i.e. by decreasing drug crystallinity [4] or crystal engineering [5-7]. Chitosan is a linear polycationic copolymer of b [1-4] linked 2-acetamido-2-deoxy-b-D-glucopyranose and 2- amino-2-deoxy-b-D-glucopyranose obtained from deacetylation of chitin, a structural polysaccharide which is very abundantly distributed in nature [8]. In recent year’s chitosan and chitosan derivatives has gained increasing interest in the pharmaceutical field due to its favorable biological properties such as biocompatibility, biodegradability, and lack of toxicity, together with its wide availability, low cost and high versatility of use [9,10] Previously chitosan was largely used as an excipient for oral drug solid dosage forms, due to its binder, anti-adherent and disintegrant properties [11-13].

Chitosan, being cationic polysaccharide in neutral or basic pH conditions, contains free amino group and hence insoluble in water. In acidic pH, amino group can undergo protonation thus making it soluble in water. It breaks down slowly to harmless products amino sugars, which are completely absorbed by the human body [14]. Chitin, a structural Moreover, its ability in improving the dissolution properties and bioavailability of poorly-soluble drugs has been proved [14-17]. Carvedilol nonselective β-adrenoreceptor blocker, chemically (±)-1-(Carbazol-4-yloxy)-3-[[2-(o-methoxyphenoxy) ethyl] amino]-2-propanol, slightly soluble in ethyl ether; and practically insoluble in water, gastric fluid (simulated, TS, pH 1.1), and intestinal fluid (simulated, TS without pancreatin, pH 7.5)

Compounds with aqueous solubility less than 1% w/v often represents dissolution rate limited absorption [18,19]. The solid systems for several drugs using chitosan have been reported with solid dispersions, co-ground mixtures and solid complexes, physical mixture and co- ground products and spray dried products at different ratios. There are reports on the improvement of dissolution rate and bioavailability of poorly water soluble drugs by precipitation of chitosan using solvent change method [20-22]. Hence objectives of present investigation were (i) To assess the feasibility of chitosan and chitosan chlorhydrate in enhancing dissolution rate of carvedilol by preparing hydrosols using solvent change method. (ii) To compare efficacy low molecular weight chitosan (chitosan chlorhydrate) and chitosan in enhancement of dissolution of carvedilol. (iii) To compare effect of different methods like solvent change approach, co- ground mixture and solid dispersions on dissolution rate of carvedilol.

## Methods

### Materials

Carvedilol was obtained as gift sample from Lupin Pharma. Ltd. Pune. India. Chitosan (85% deactylated), chitosan chlorhydrate obtained from mehtani chitosan India. Hydrochloric acid (35-38%), glacial acetic acid (99.5%), sodium citrate (99.5%) was purchased from Loba chemicals. Mumbai, India. All other chemicals were analytical grade.

### Methods

#### Preparation of hydrosols

The composition of different batchhes is given in Table 1. Chitosan solution was prepared by soaking chitosan and chitosan chlorhydrate in 1% glacial acetic acid for 3 hrs. a weighed amount of drug was dispersed in chitosan solution by stirring at 4000 rpm for 25 min. then dispersion was added to sodium citrate solution to precipitate chitosan on drug to form hydrosols. The precipitate obtained was filtered through Whatmann No. 1 filter paper using vacuum filtration unit and dried at 45°C for 24 h. the dried product then passed through sieve No. 60 to obtain uniform size distribution. A control hydrosol formulation without chitosan was also prepared (C12) (Table 1). The practical yield of prepared hydrosols was calculated.

**Table 1:** Composition of carvedilol- chitosan and chitosan chlorhydrtate hydrosol formulations

**Batch code**	**Carvedilol (mg)**	**Chitosan (%)**	**Chitosan chlorhydrate (%)**	**Glacial acetic acid (ml)**	**Sodium citrate (2% w/v) (ml)**
C1	500	0.05	-	20	100
C2	500	0.2	-	20	100
C3	500	0.4	-	20	100
C4	500	0.6	-	20	100
C5	500	1	-	20	100
C6	500	-	0.05	20	100
C7	500	-	0.2	20	100
C8	500	-	0.4	20	100
C9	500	-	0.6	20	100
C10	500	-	0.8	20	100
C11	500	-	1	20	100
C12	500	-	-	20	100

#### Preparation of co-grind mixture

The composition of different batches is given in Table 2. Listed amount of carvedilol was accurately weighed and co grinded with chitosan and chitosan chlorhydrate in mortar for 30 min. Product then passed through sieve No. 60 to obtain uniform size distribution. A control formulation without chitosan was also prepared (C24) (Table 2).

**Table 2:** Composition of carvedilol - chitosan and chitosan chlorhydrtate cogrind mixture

**Batch code**	**Carvedilol (mg)**	**Chitosan (mg)**	**Chitosan chlorhydrate (mg)**
C13	500	5	-
C14	500	20	-
C15	500	40	-
C16	500	60	-
C17	500	100	-
C18	500	-	5
C19	500	-	20
C20	500	-	40
C21	500	-	60
C22	500	-	80
C23	500	-	100
C24	500	-	-

#### Preparation of solid dispersions

The composition of different batches is given in Table 3. Chitosan solution was prepared by soaking chitosan and chitosan chlorhydrate in 1% glacial acetic acid for 3 hrs. A weighed amount of drug was dispersed in chitosan solution by stirring at 4000 rpm for 25 min, and then dispersion was dried at 45°C for 24 hrs. The dried product then passed through sieve No. 60 to obtain uniform size distribution. A control dispersion formulation without chitosan was also prepared (C36) (Table 3).

**Table 3:** Composition of carvedilol- chitosan and chitosan chlorhydrtate solid dispersions

**Batch code**	**Carvedilol (mg)**	**Chitosan (%)**	**Chitosan chlorhydrate (%)**	**Glacial acetic acid (ml)**
C25	500	0.05	-	20
C26	500	0.2	-	20
C27	500	0.4	-	20
C28	500	0.6	-	20
C29	500	1	-	20
C30	500	-	0.05	20
C31	500	-	0.2	20
C32	500	-	0.4	20
C33	500	-	0.6	20
C34	500	-	0.8	20
C35	500	-	1	20
C36	500	-	-	20

#### Solubility, drug content determination

An excess amount of carvedilol or prepared formulations was placed in the vials containing 10 ml of different solvents (water and simulated gastric fluid (SGF) without enzymes pH 1.2) the vials were agitated in a incubator shaker (100 rpm/min) for 24 hrs. The solution was filtered through a membrane filter (0.45 μm) the amount of drug solublized was analyzed spectrophotometrically (JASCO, V-530, Japan) at 241.5 nm. This study was carried out to determine the saturation solubility of carvedilol in water and SGF.

For determination of drug content, prepared hydrosols, solid dispersions and co-ground mixture (10 mg) were triturated with SGF and finally volume was made up to 100 ml with the same. The solution was filtered through a membrane filter (0.45 μm) the amount of drug solublized was analyzed spectrophotometrically (JASCO, V-530, Japan) at 241.5 nm after sufficient dilutions with SGF.

#### Infrared (IR) spectroscopy

IR spectroscopy was conducted using a Shimadzu FTIR 8300S Spectrophotometer (Shimadzu, Tokyo, Japan) and the spectrum was recorded in the wavelength region of 4000–400 cm^−1^. The procedure consisted of dispersing a sample (drug, mixture of drug and polymer and prepared hydrosols) in KBr and compressing into discs by applying a pressure of 5 t for 5 min in a hydraulic press. The pellet was placed in the light path and the spectrum was recorded. All spectra were collected as an average of three scans at a resolution of 2 cm^−1^.

#### Differential scanning colorimetry (DSC)

DSC was performed using DSC-60A (Shimadzu, Tokyo, Japan) calorimeter to study the thermal behavior of drug alone and prepared co-hydrosols. The samples were heated in hermetically sealed aluminum pans under nitrogen flow (30 ml/min) at a scanning rate of 10°C/min from 50°C to 300°C. Empty aluminum pan was used as a reference. The physical mixture of drug with excipients for compatibility studies was prepared by triturating the drug with excipients in a dried mortar for 5 min.

#### Powder X- Ray diffraction (PXRD)

The X-ray diffraction patterns of pure drug and the optimized hydrosols formulation were recorded using PHilips analytical X-ray diffractometer (Model: PW 3710) (PHilips, Almelo, The Netherlands) with a copper target over the interval of 5–70° 2θ^-1^. The conditions were: voltage 40 kV; current 30 mA; scanning speed 2◦/min; temperature of acquisition: room temperature; detector: scintillation counter detector; sample holder: non-rotating holder.

#### Electron microscopy (EM)

The surface characteristics of the pure drug and prepared hydrosols were studied by binocular EM (NIKON, Tokyo, Japan) at 100 × .

#### *In vitro* dissolution study

The dissolution rate of Carvedilol alone and prepared formulations were measured in triplicate in a dissolution apparatus (Lab India, Model Disso 2000 Tablet dissolution test apparatus, Mumbai, India) using apparatus USP Type II. Dissolution studies were carried out using 900 mL SGF (Simulated gastric fluid pH 1.2) at 37° ± 0.5°C at 50 rpm. 30 mg carvedilol or its equivalent amount formulations were placed in 900 mL of SGF. 5 mL samples were withdrawn after 15, 30, 45 min. Replaced each time with 5 mL fresh SGF to maintain a proper sink condition. The solutions were immediately filtered through 0.45 μm membrane filter, diluted and the concentration of carvedilol was determined spectro-photometrically at 241.5 nm.

#### Stability study

After determining the drug content, the optimized formulations were charged for accelerated stability studies as per ICH guidelines. The samples (each 100 mg, n = 3) were kept for stability studies at 40 ± 2°C and 75 ± 5% RH for a period of 6 months in environmental test chamber (HMG INDIA, Mumbai). The samples were kept in glass vials sealed with rubber plugs. 10 mg of stored hydrosols were taken out at 15, 30, 60, 90 and 180 days, they were analyzed for drug content and physical change.

## Results and discussion

The solubility, dissolution behavior and permeability of a drug are the key determinants of its oral bioavailability. The solubility data of Carvedilol reveals that it is poorly soluble in water. Therefore, the improvement of carvedilol dissolution from its oral solid dosage forms is of great concern. The practical yield in case of hydrosols was ranged from 59.76 to 92.32% (Table 4).

**Table 4:** Practical yield, drug content and solubility data in water and SGF for the pure drug and prepared hydrosols, cogrind mixture and solid dispersions

**Formulation code**	**% Yield**	**Drug content**	**Water**	**SGF**
			**(mg/ml)**	**(mg/ml)**
Carvedilol	-	-	0.13 ± 0.002	0.43 ± 0.001
C1	85.62	99.23 ±0.58	0.21 ± 0.001	0.41 ± 0.004
C2	80.32	99.12 ± 0.76	0.37 ± 0.002	0.39 ± 0.004
C3	75.86	99.45 ± 0.68	0.39 ± 0.004	1.2 ± 0.002
C4	64.83	99.78 ± 0.98	0.36 ± 0.004	1.03 ± 0.002
C5	59.76	98.24 ± 1.23	0.35 ± 0.001	1.04 ± 0.004
C6	86.86	98.45 ± 0.98	0.31 ± 0.005	1.00 ± 0.004
C7	82.23	99.14 ± 1.45	0.72 ± 0.001	0.86 ± 0.001
C8	73.32	99.56 ± 1.02	0.74 ± 0.005	0.41 ± 0.001
C9	69.46	99.89 ± 1.13	0.60 ± 0.005	0.42 ± 0.006
C10	63.23	99.25 ± 0.87	0.54 ± 0.005	0.41 ± 0.001
C11	59.21	98.78 ± 0.96	0.68 ± 0.004	0.42 ± 0.002
C12	93.32	99.63 ± 1.96	0.14 ± 0.001	0.41 ± 0.004
C13	100	98.32 ± 1.06	0.43 ± 0.002	0.42 ± 0.007
C14	100	99.32 ± 1.23	0.78 ± 0.001	0.45 ± 0.004
C15	100	98.65 ± 0.98	2.36 ± 0.004	0.46 ± 0.003
C16	100	99.12 ± 1.24	1.2 ± 0.001	0.55 ± 0.004
C17	100	98.56 ± 0.91	1.40 ± 0.001	0.46 ± 0.002
C18	100	99.12 ± 0.85	0.45 ± 0.002	0.41 ± 0.003
C19	100	98.95 ± 0.89	1.23 ± 0.004	0.42 ± 0.002
C20	100	99.12 ± 0.92	2.01 ± 0.004	0.50 ± 0.004
C21	100	99.17 ± 1.44	3.21 ± 0.001	0.33 ± 0.008
C22	100	98.32 ± 1.56	2.14 ± 0.006	0.40 ± 0.002
C23	100	99.32 ± 0.97	3.45 ± 0.003	0.33 ± 0.001
C24	100	99.10 ± 1.12	0.23 ± 0.002	0.43 ± 0.002
C25	98.35	98.56 ± 0.97	0.42 ± 0.003	0.46 ± 0.004
C26	99.56	99.45 ± 1.35	0.78 ± 0.004	0.47 ± 0.001
C27	99.00	99.78 ± 1.65	1.38 ± 0.004	0.36 ± 0.002
C28	99.23	99.87 ± 0.94	1.53 ± 0.001	0.45 ± 0.002
C29	99.65	98.92 ± 0.96	0.97 ± 0.004	0.47 ± 0.004
C30	98.32	98.23 ± 0.98	0.26 ± 0.002	0.48 ± 0.005
C31	99.32	99.15 ± 0.0.91	0.54 ± 0.003	0.45 ± 0.007
C32	99.23	99.25 ± 1.25	0.65 ± 0.001	0.40 ± 0.003
C33	99.78	99.42 ± 1.32	0.68 ± 0.002	0.47 ± 0.002
C34	99.65	99.89 ± 1.20	0.89 ± 0.004	0.48 ± 0.004
C35	99.21	99.47 ± 1.02	1.62 ± 0.002	0.42 ± 0.003
C36	98.96	99.56 ± 1.05	0.13 ± 0.006	0.42 ± 0.004

The practical yield was found to be decreased with the increase in polymer concentration due to the formation of a thick viscous chitosan solution from which separation of the drug hydrosols was difficult. The drug content was found to be good and uniform among the different batches of hydrosols, cogrind mixture and solid dispersions ranged from 98.24 to 99.89% (Table 4). The solubility studies of the chitosan cogrind mixture, C-15 showed highest solubility of drug in both water (2.36 mg/ml) in comparison with pure drug (0.13 mg/ml). If we compare solubility data of chitosan and chitosan chlorhydrtae batch C21 showed better aqueous solubility (3.21 mg/ml). No difference was observed in case of solubility in simulated gastric fluid. The difference in aqueous solubility of chitosan and chitosan chlorhydrate may be due to better enhanced wetting property due to salting out, low molecular weight of chitosan chlorhydrate. In addition, as the concentration of chitosan or chitosan chlorhydrtae increased in the formulation, the solubility gradually increased up to a certain concentration followed by decrease in the solubility. If we compare the methods of formulation co-grind mixture showed higher solubility data than solid dispersions and hydrosols, this might due to micronization or sustained supersaturation of material in media for cogrind mixture.

**Figure 1:**
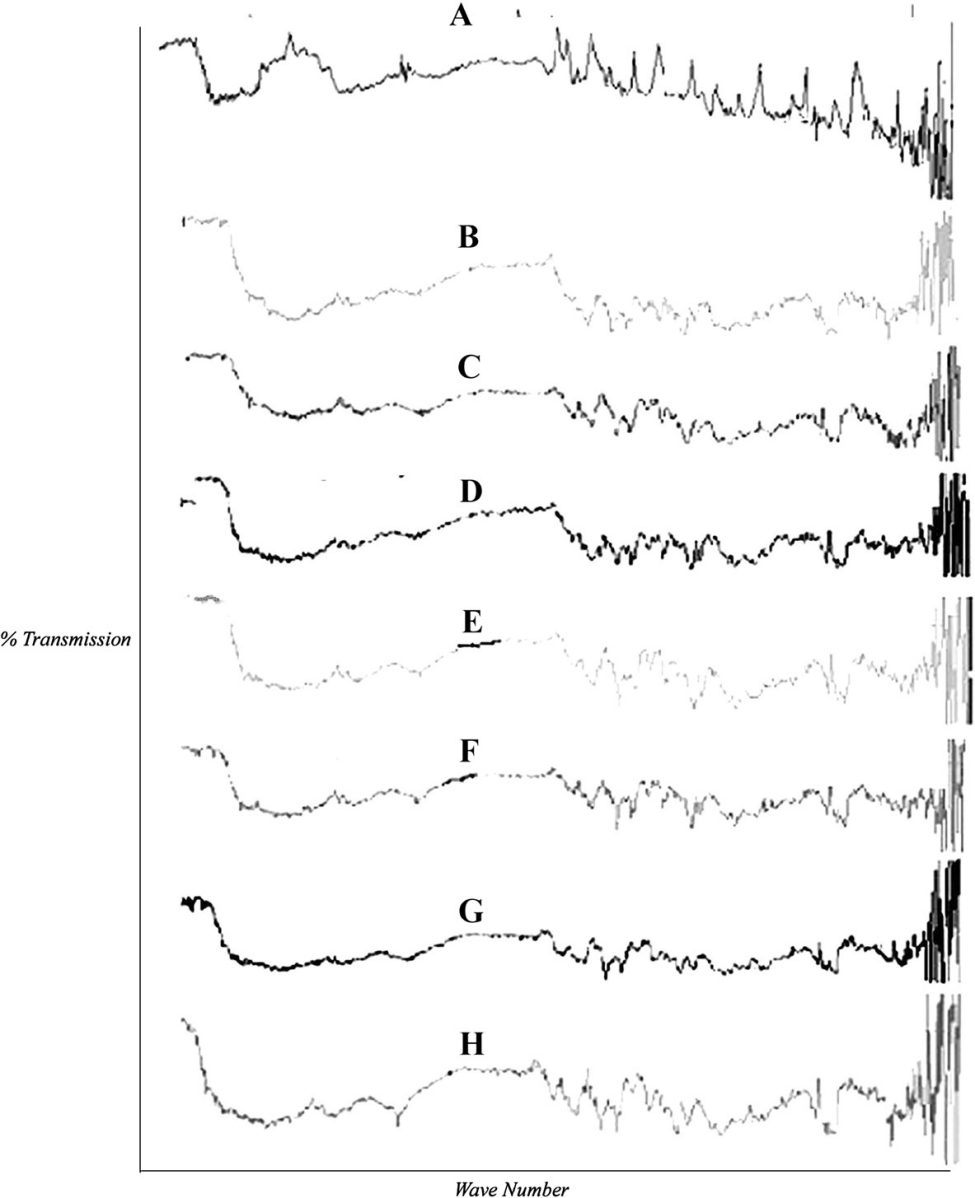
**Comparative FTIR pattern of carvedilol and Chitosan based hydrosols, solid dispersions and co grinding mixture.A**. chitosan, **B**. Carvedilol, **C**.0.2% chitosan hydrosols, **D**. 0.4% Ch-chlorhydrate hydrosols, **E**. SD 0.4% chitosan, **F**. SD 0.8% Ch-chlorhydrate, **G**. 0.4% chitosan cogrind mixture, **H**. 0.4% ch-chlorhydrate cogrind mixture.

The possible interaction between drug and chitosan as hydrosol formers were studied by IR spectroscopy (Figure 1). The pure carvedilol showed peaks at 3300–3500 cm^-1^, onset around 1600 cm^-1^, other around 1500 cm^-^1, 1000–1300 cm^-1^ due to presence of N-H stretch, O-H stretch, consistent with aromatic compounds and C-OH stretch respectively. It was observed that all important peaks due to functional group of drug are present in the hydrosols along with some new intense peaks indicating the presence of hydrogen bonding. Also there are peaks observed in the range of 400–800 cm^-1^ in hydrosols prepared from chitosan chlorhydrate indicative of halogen hydrogen interaction.

**Figure 2:**
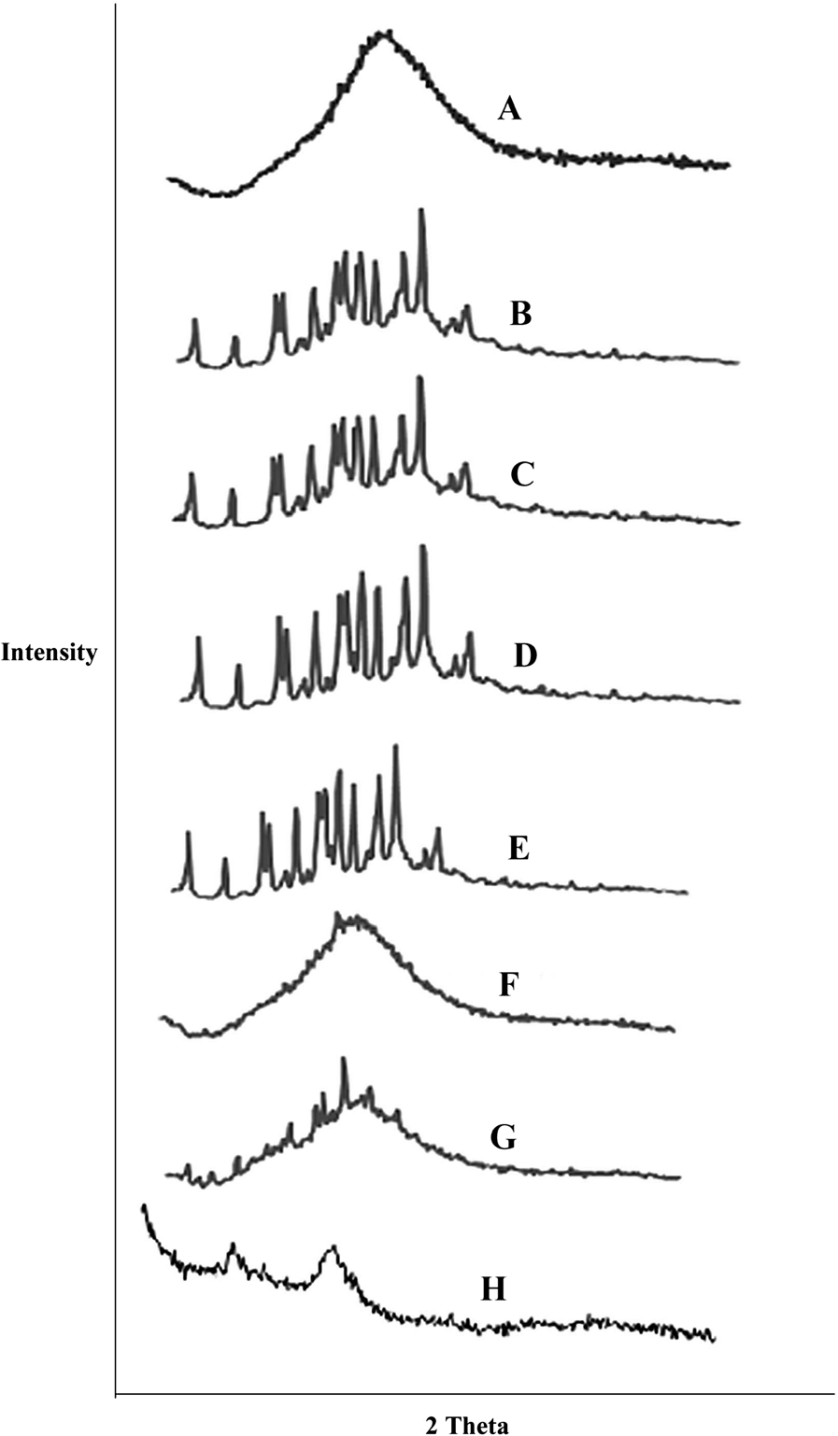
**Comparative P-XRD pattern of carvedilol and Chitosan based hydrosols, solid dispersions and co grinding mixture.A**. Carvedilol, **B**. 0.2% chitosan hydrosols, **C**. 0.4% Ch-chlorhydrate hydrosols, **D**. SD 0.4% chitosan, **E**. SD 0.8% Ch-chlorhydrate, **F**. 0.4% chitosan cogrind mixture, **G**. 0.4% ch-chlorhydrate cogrind mixture, H. chitosan.

The PXRD patterns of the pure drug and the hydrosols are shown in Figure 2. The XRD scan of carvedilol showed intense peak of cystallanity at 22.7° (2θ) with peak intensity of 1832 indicating its crystalline nature. The relative degree of crystllanity values of carvedilol hydrosols at specific angle are 1.22, 1.08, 1.18, 0.833, 1.04, 1.00 for prepared chitosan and chitosan chlorhydrate hydrosols as shown in Figure 2 respectively. It indicates crystalline nature of carvedilol in prepared hydrosols.

**Figure 3:**
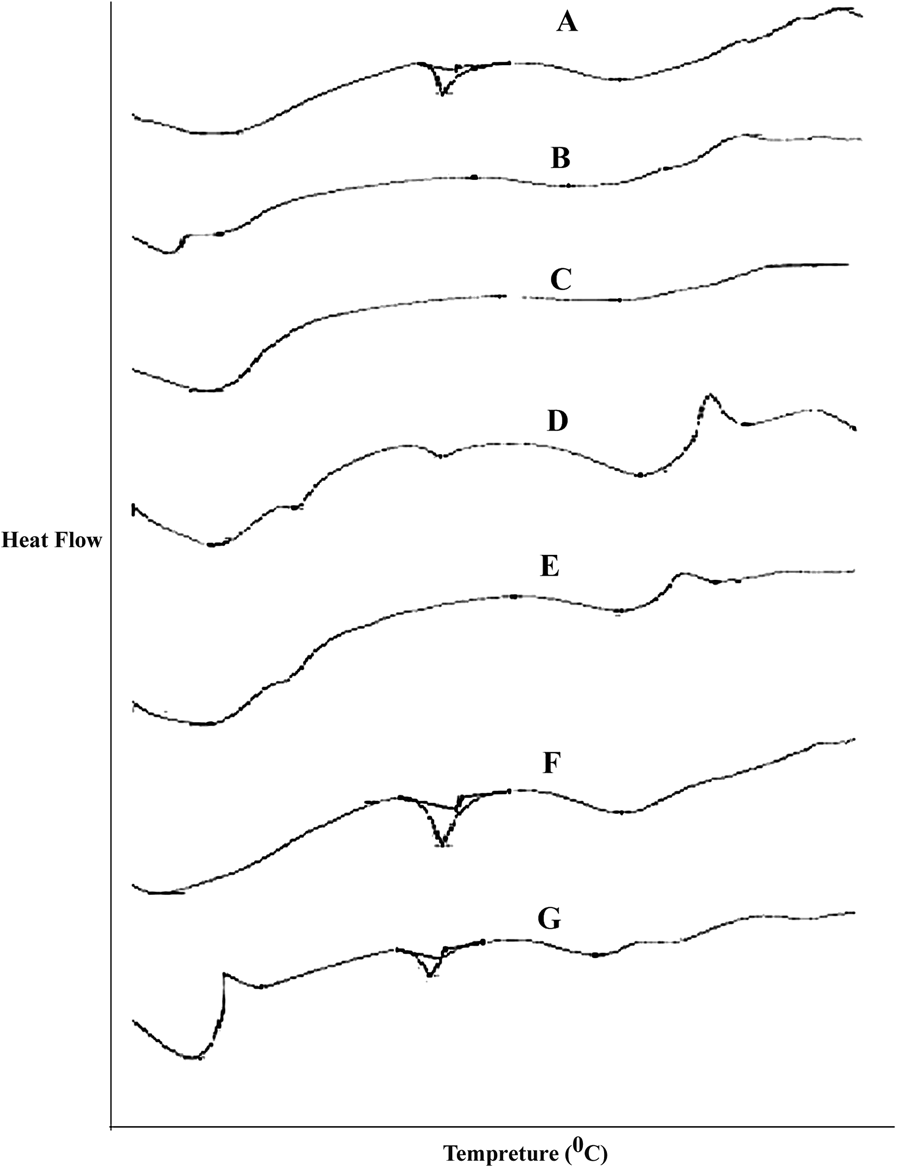
**Comparative DSC of carvedilol and formulations A.** Carvedilol, **B**. 0.2% Chitosan hydrosols, **C**. 0.4% Chitosan chlorhydrate hydrosols, **D**. 0.2% Chitosan solid dispersions, **E**. 0.8% Chitosan chlorhydrate solid dispersions, **F**. 0.4% Chitosan Co-grinding, **G**. 0.4% Chitosan chlorhydrate Co-grinding.

The results of DSC studies are given in Figure 3 and Table 5. Pure carvedilol showed a sharp endotherm at 155.94°C corresponding to its melting point. There was no appreciable change in the melting endotherms of the cogrind mixture as compare pure drug. However there is disappearance of melting endothermic peaks of carvedilol when formulated into hydrosols and solid dispersions meaning is that drug has been converted into amorphous form and chitosan and chitosan chlorhydtare in crystalline form. The endothermic peaks for chitosan and chitosan chlorhydrate were observed in the range of 200–225°C.

**Table 5:** Melting endotherms of carvedilol and prepared formulations

**Formulations**	**Melting endotherm at (°C)**
Carvedilol	**155.94**
0.2% Chitosan hydrosols	**Nil**
0.4% Chitosan chlorhydrate hydrosols	**Nil**
0.2% Chitosan solid dispersions	**Nil**
0.8% Chitosan chlorhydrate solid dispersions	**Nil**
0.4% Chitosan Co-grinding	**153.78**
0.4% Chitosan chlorhydrate Co-grinding	**152.97**

**Figure 4:**
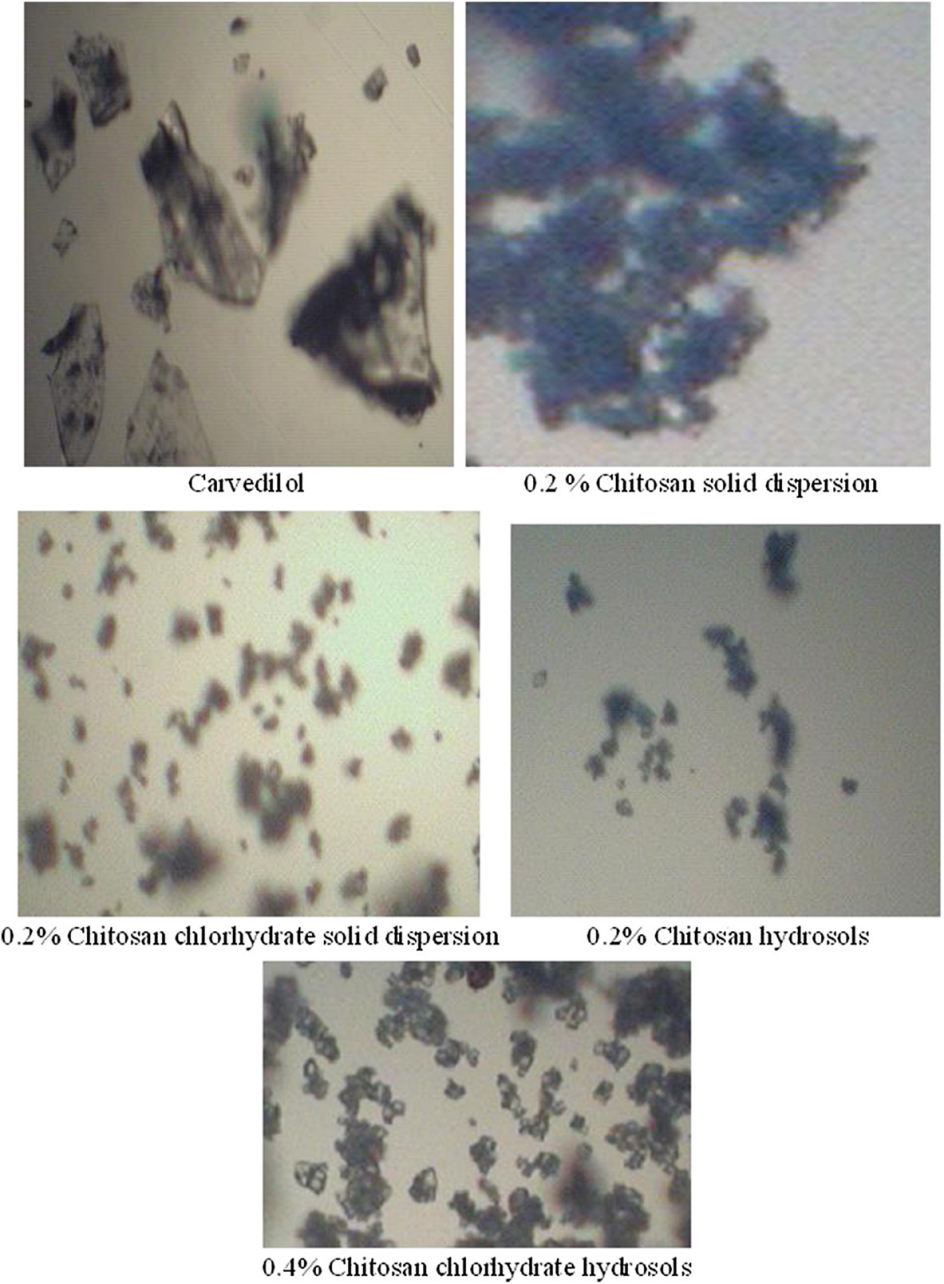
Electronic microphotographs of optimized formulations.

The photomicrographs of pure carvedilol and the selected crystal formulations are given in Figure 4. The pure carvedilol was characterized by hydrosols of bigger size and regular shape with an apparently smooth surface. In contrast hydrosols were present in the form of fine powder. Additionally, solid dispersions were porous and with rough surface which might have resulted in the enhanced dissolution rate as compared to pure drug. The size of hydrosols was comparatively less than carvedilol.

**Figure 5:**
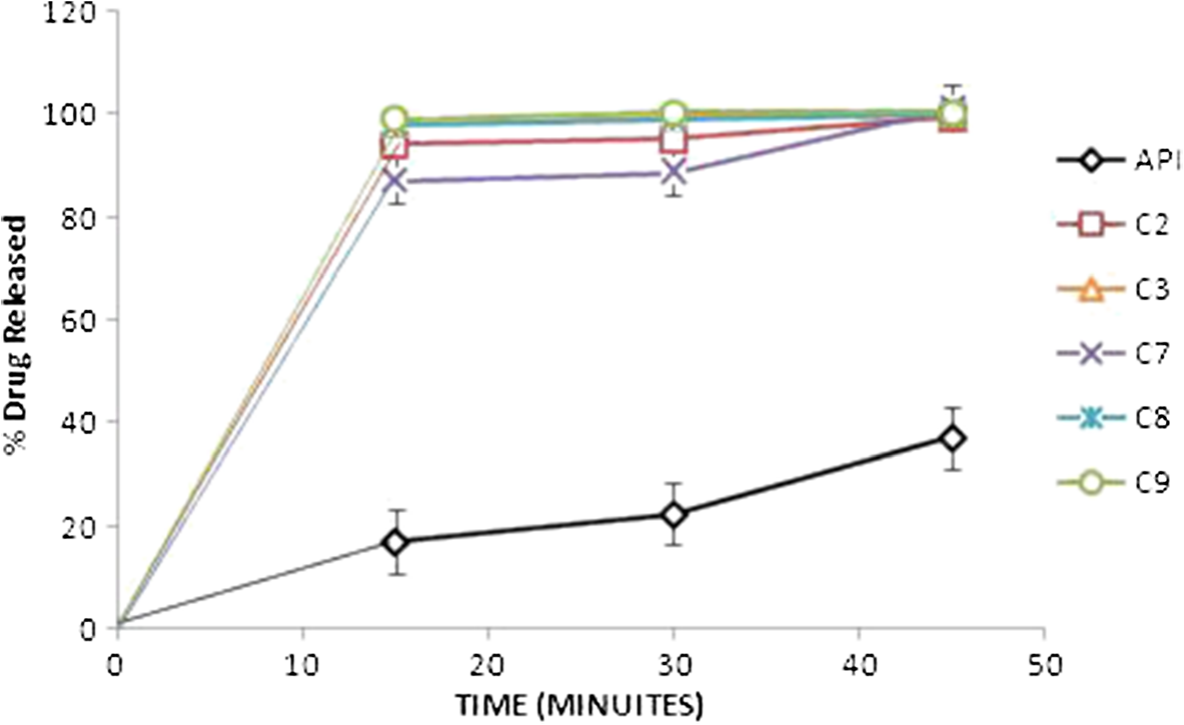
***In vitro *release of carvedilol from the prepared hydrosol formulations in SGF.** The points represent mean values, n = 3.

**Figure 6:**
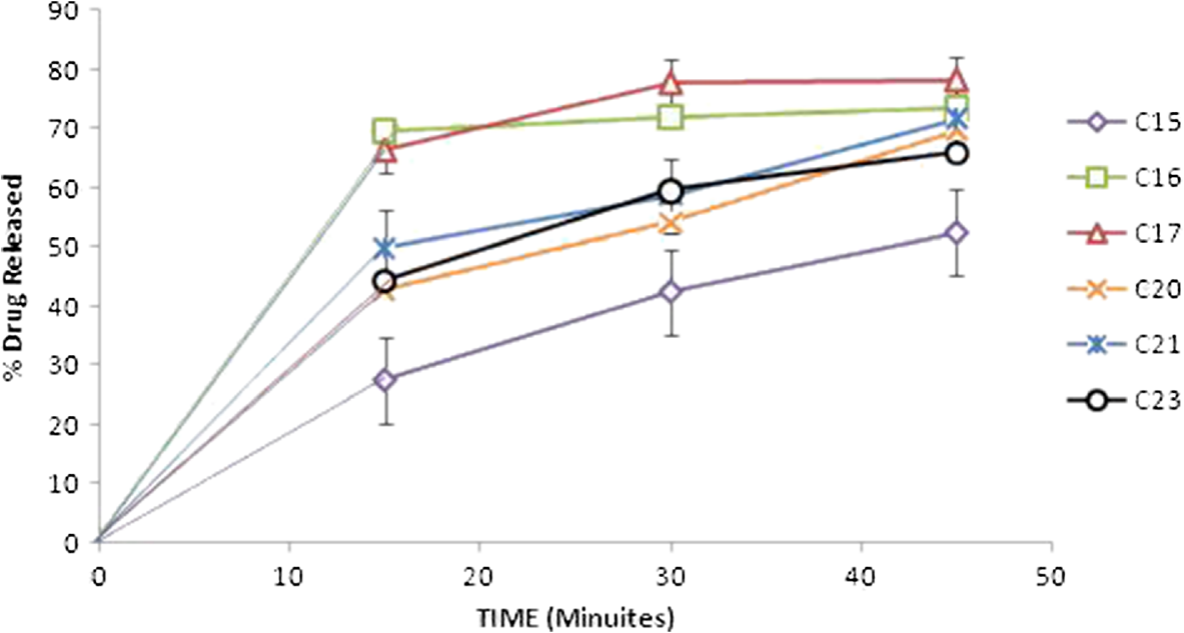
**In vitro release of carvedilol from the prepared cogrind formulations in SGF.** The points represent mean values, n = 3.

The increase in the carvedilol solubility, although little, and dramatically increase in its dissolution rate from prepared hydrosols, cogrind mixture and solid dispersions can be explained as follow (as shown in Figures 5 &6):

Chitosan has been proposed as a useful excipient for enhancing the bioavailability of poorly water-soluble compounds [23]. Chitosan and its derivatives have been reported as a good vehicle for enhancing the solubility and dissolution of poorly water-soluble drugs [24-26]. Chitosan dissolves readily in most of the acid solutions and upon dissolution, amine groups of the polymer become protonated, resulting in a positively charged polysaccharide (RNH3+) and chitosan salts (chloride, glutamate, etc.) that are soluble in water [27].

The earlier literature reveals that dissolution rate not only depends on the surface area and particle size of the processed powder, but is greatly affected by crystal morphhology and wettability [27] So increased wettability of the drug by the adsorption of chitosan and chitosan chlorhydrate onto the hydrophobic surface of the drug is the first reason. In a previous study also, chitosan showed increased solubility and dissolution rate of naproxen due to adsorption on its surface [26]. One interesting case was observed all solid dispersions showed 100% drug release within 15 minutes. Carvedilol exhibited pH dependent solubility. Its solubility decreases with increasing pH then starts decreasing after pH 4. At lower pH values protonated form of carvedilol and its salts generated *in situ* will determine its solubility. Hydrochloride salt generated *in situ* in an acidic medium might be less soluble in this medium than the protonated carvedilol itself. At basic pH as the pH increases from 9.2 to 11 its solubility remains more or less constant [28]. In formulation of solid dispersions glacial acetic acid was used so acetic acid incorporates acidity to the formulation that’s why there might be possibility of enhancement of dissolution rate of carvediol [28].

The difference in dissolution rate enhancement by chitosan and chitosan chlorhydrate can be explained as, the trend found was that for the two types of chitosan (base and salt), the lower the *M*w, the faster was the drug dissolution. This behavior was predictable taking into account the relationship between *M*w and viscosity of polymer solution [29].

Upon contact with the acidic medium; chitosan swells and forms a gel. The diffusion of the drug through the gel into the release medium would be retarded by increasing the viscosity of the polymer, and hence of the gel [30]. On the other hand, the chitosan chlorhydrate led to faster drug dissolution than chitosan. The explanation to this behavior was found in the differences in the wetting rate, solubilities and swelling capacity of the chitosan and chitosan salts, chitosan chlorhydrate rapidly wet and dissolve upon its incorporation into the dissolution medium, whereas the CS base, less water soluble, would take more time to dissolve. In order to clarify the causes of significant difference in the dissolution rate, the surface morphology of the hydrosols was examined by electronic microscope (EM). Thus the fine and fluffy physical state of C-3 hydrosols along with their porous and rough surface as supported by EM might also have contributed to the enhanced solubility and dissolution rate of carvedilol from these hydrosols.

The physical stability of optimized formulations was compared with the drug. The formulations were found to be stable during the study periods there was no any change in color was found. The drug content in all the formulations was found to be within the limit. The drug content within the formulations is shown in the Table 6.

**Table 6:** Drug content of formulations after stability studies

**Storage condition 30 ± 2°C/65 ± 5% RH**	**Drug Content (%) of formulations**						
	**Carvedilol**	**0.2% Chitosan hydrosols**	**0.4% Chitosan chlorhydrate hydrosols**	**0.2% chitosan solid dispersions**	**0.8% chitosan chlorhydrate solid dispersions**	**0.4% Chitosan Co-grinding**	**0.4% Chitosan chlorhydrate Co-grinding**
**Time 0 days**	99.56 ± 1.16	98.77 ± 1.31	98.19 ± 2.13	99.66 ± 1.24	98.17 ± 1.14	99.46 ± 1.11	98.86 ± 1.15
**Time 90 days**	99.46 ± 1.38	98.67 ± 1.44	98.09 ± 2.34	99.76 ± 1.32	98.67 ± 1.43	99.27 ± 1.12	98.66 ± 1.34
**Time 180 days**	99.26 ± 1.10	98.47 ± 1.22	97.69 ± 2.01	99.16 ± 1.16	98.97 ± 1.25	99.29 ± 1.14	99.10 ± 1.17

## Conclusions

The present study investigated successful and simple methods to enhance the dissolution rate of carvedilol by using chitosan and chitosan chlorhydrate. If this process can be scaled-up to manufacturing level, this technique has the potential to develop into an invaluable technology in future.

## Competing interests

The authors report no competing interests. The authors alone are responsible for the content and writing of the paper.

## Authors’ contributions

SAS: carried out the formulation studies, characterized the samples for evaluation parameters participated in the sequence alignment and drafted the manuscript. YAV participated in sequence alignment. MSM participated in its design and coordination and helped to draft the manuscript and interpretation of spectral analysis. All authors read and approved the final manuscript.
